# Supplementary material for: Exploring spatial variation and multilevel modeling of malaria prevalence among children aged 6-59 months based on RDT in Niger: Insights for public health decision-making
Source: PLoS One. 2025 Dec 1;20(12):e0336022. doi: 10.1371/journal.pone.0336022 (PMC12668621; doi:10.1371/journal.pone.0336022)
Supplement: Attachment — Submitted filename: Review Dr Bougouma Edith 06 07 2025.docx [file pone.0336022.s001.docx]

1. Recommendation (Answer options: Accept, Minor Revision, Major Revision, Reject)

Final recommendation: Acceptance with minor revisions

The article is solid, well-structured and relevant for the scientific community and public health decision-makers. A few methodological clarifications and a slight lightening of the presentation of results will enhance its readability.

2. Is the manuscript technically sound, and do the data support the conclusions? (Answer options: Yes, No, Partly)

Yes

3. Has the statistical analysis been performed appropriately and rigorously? (Answer options: Yes, No, I don’t know, N/A)

Yes

4. Does the manuscript adhere to the PLOS Data Policy? Additional details can be found at http://journals.plos.org/plosone/s/materials-and-software-sharing. (Answer options: Yes, No)

Yes

5. Is the manuscript presented in an intelligible fashion and written in standard English? (Answer options: Yes, No)

Yes

6. Review Comments to the Author (minimum 200 characters)

1. Scientific relevance and originality

The study addresses a relevant and major public health issue in sub-Saharan Africa: the prevalence of malaria in children under five. The combined approach of spatial analysis and multilevel modeling is relevant and well justified. The originality lies in the use of NMIS 2021 data to produce a fine mapping of at-risk areas and identify multi-level determinants.

- Assessment: Relevant subject, innovative methodological approach for the Niger context.

2. Methodology

The article uses robust methods:

- Spatial analysis (Moran's I, Getis-Ord Gi*, SaTScan)

- Geostatistical interpolation (Empirical Bayesian Kriging)

- Multilevel modeling (mixed-effects logistics)

The models are well described, with goodness-of-fit indicators (AIC, BIC, DIC, ICC, MOR, PCV). Stratification of variables at different levels (individual, household, community) is rigorous.

- Suggestions:

- Clarify certain methodological choices, in particular the significance threshold for spatial clusters.

- Further justify the interpretation of certain counter-intuitive associations (e.g. non-anemia associated with higher prevalence).

3. Results

The results are clearly presented, with detailed tables and relevant geographical figures. The identification of high-risk clusters in the Zinder, Dosso, Maradi and Tillabéri regions is particularly useful for decision-makers.

- Strengths:

- Good visualization of spatial data.

- Well-structured multi-level analysis.

- Areas for improvement:

- Summarize key results more in the text to avoid number overload.

- Standardize percentages and headcounts in tables.

4. Discussion and interpretation

The discussion is well grounded in the existing literature. The authors interpret the results with caution and highlight the implications for public health policy.

- Recommendations:

- Deepen discussion of regional disparities and structural factors (access to care, infrastructure).

- Incorporate consideration of the limitations of RDTs as a diagnostic tool (sensitivity, specificity).

5. Conclusion

The conclusion is consistent with the results and highlights concrete recommendations: increased coverage of impregnated mosquito nets, targeted interventions according to risk areas, and the importance of maternal education.

- Assessment: Relevant conclusion, well aligned with study objectives.

6. Ethics, data availability and conflicts of interest

- The authors state that the data come from the DHS Program and are publicly available.

- No conflicts of interest declared.

- No direct collection of human data: ethics respected.

7. PLOS authors have the option to publish the peer review history of their article (https://journals.plos.org/plosone/s/editorial-and-peer-review-process#loc-peer-review-history). If published, this will include your full peer review and any attached files. Do you want your identity to be public for this peer review? (Answer options: Yes, No)

Yes

7a. If you answered "Yes" above, that you would like your identity to be public, please provide your full name, as it should appear on the published peer review. Please do not sign the review on behalf of another person.

**Dr Bougouma Edith Christiane /** [**Bougouma.cedith@gmail.com**](mailto:Bougouma.cedith@gmail.com)

**Confidential to Editor :**

1. Do you have any potentially competing interests? If none, type "None." Our policy on competing interests can be found at http://journals.plos.org/plosone/s/competing-interests.

"**None."**

2. Did you receive any assistance in preparing this review (e.g. from a post-doc or graduate student)? If yes, please provide their name and title. If you have chosen to reveal your identity to the authors in question 7 above, please include the name of the person who assisted you in the response to question 7 as well. (optional)

**"None."**

3. Do you want to receive recognition for this review on a Web of Science researcher profile? (https://clarivate.com/products/scientific-and-academic-research/research-discovery-and-workflow-solutions/researcher-profiles/)? If you opt in, your Web of Science profile will automatically be updated to show a verified record of this review in full compliance with the journal's review policy. If you don't have a Web of Science profile, you will need to first create a free account. (Answer options: Yes, No)

**Yes**
