# Supplementary material for: Exploring spatial variation and multilevel modeling of malaria prevalence among children aged 6-59 months based on RDT in Niger: Insights for public health decision-making
Source: PLoS One. 2025 Dec 1;20(12):e0336022. doi: 10.1371/journal.pone.0336022 (PMC12668621; doi:10.1371/journal.pone.0336022)
Supplement: Attachment — Submitted filename: Response to Reviewers.docx [file pone.0336022.s002.docx]

PONE-D-25-18518

**Exploring Spatial Variation and Multilevel Modeling of Malaria Prevalence among Children Aged 6-59 Months Based on RDT in Niger: Findings from the 2021 NMIS**

PLOS ONE

Manuscript Comment and Correction

| 1. Please ensure that your manuscript meets PLOS ONE's style requirements, including those for file naming. The PLOS ONE style templates can be found at  <https://journals.plos.org/plosone/s/file?id=wjVg/PLOSOne_formatting_sample_main_body.pdf> and  [https://journals.plos.org/plosone/s/file?id=ba62/PLOSOne_formatting_sample_title_authors_affiliations.pdf](https://track.editorialmanager.com/CL0/https:%2F%2Fjournals.plos.org%2Fplosone%2Fs%2Ffile%3Fid=ba62%2FPLOSOne_formatting_sample_title_authors_affiliations.pdf/1/010f01992eb25359-1c96c826-1c47-420f-8556-9f5c4ef5464a-000000/O-wSPiSkQ7EWVhvthNnxssFYPk9HLmla30XnlJZG8JE=227) | We have corrected the order of authors and updated their affiliations in accordance with the PLOS Author and Affiliation formatting guidelines. |
| --- | --- |
| 2. Please include a separate caption for each figure in your manuscript. | We have prepared and included a separate caption for each figure in the revised manuscript. |
| 3. We note that Figures 2, 3, and 4 in your submission contain [map/satellite] images which may be copyrighted. All PLOS content is published under the Creative Commons Attribution License (CC BY 4.0), which means that the manuscript, images, and Supporting Information files will be freely available online, and any third party is permitted to access, download, copy, distribute, and use these materials in any way, even commercially, with proper attribution. For these reasons, we cannot publish previously copyrighted maps or satellite images created using proprietary data, such as Google software (Google Maps, Street View, and Earth). For more information, see our copyright guidelines: <http://journals.plos.org/plosone/s/licenses-and-copyright>. | We confirm that Figures 2, 3, and 4 were created entirely by the authors based on publicly available shapefiles and survey cluster data from the 2021 Niger Malaria Indicator Survey (NMIS) using ArcGIS software, based on publicly available shapefiles/data (not from copyrighted sources such as Google Maps, Earth, or Street View). The figures are original works, and copyright rests with the authors. We agree to have them published under the Creative Commons Attribution License (CC BY 4.0), and they may therefore be freely shared, distributed, and reused with proper attribution. |
| 4. If the reviewer comments include a recommendation to cite specific previously published works, please review and evaluate these publications to determine whether they are relevant and should be cited. There is no requirement to cite these works unless the editor has indicated otherwise. | There is no such kind of recommendations from the reviewer’s side. |
| 5. Please review your reference list to ensure that it is complete and correct. If you have cited papers that have been retracted, please include the rationale for doing so in the manuscript text, or remove these references and replace them with relevant current references. Any changes to the reference list should be mentioned in the rebuttal letter that accompanies your revised manuscript. If you need to cite a retracted article, indicate the article’s retracted status in the References list and also include a citation and full reference for the retraction notice. | The reference list has been reviewed and is correct as submitted. |
| **5. Review Comments to the Author**  **Reviewer #1:** | |
| 2. Methodology  ϖ Suggestions:  - Clarify certain methodological choices, in particular the significance threshold for spatial clusters. - Further justify the interpretation of certain counter-intuitive associations (e.g. non-anemia associated with higher prevalence). | We have made revision with significance threshold details in separate section and define **how significance was assessed** in Moran’s I, Gi*, and SaTScan.  We appreciate the reviewer’s observation regarding the counter-intuitive finding. Several possible explanations may account for our result. We have revised the discussion section to include these points and provide a more balanced interpretation. |
| 3. Results  ϖ Areas for improvement:  - Summarize key results more in the text to avoid number overload.  - Standardize percentages and headcounts in tables. | -Thank you for your valuable feedback on the table's structure. As per your recommendation, we have revised the table to ensure all data is presented in a standardized format. Specifically, we have implemented a uniform basis for all percentage calculations, using a single, consistent denominator across the entire table.  -We appreciate the feedback regarding the need to summarize our key results more concisely to avoid 'number overload.' Given the nature of our spatial modeling, which generates extensive data, we have carefully selected only the most relevant and essential numerical figures for inclusion in the main text. Presenting these specific values is crucial, as further removal would render the analysis less informative and compromise the integrity of our findings. Therefore, we have opted to prioritize a comprehensive presentation of our results over a text-heavy narrative, ensuring the reader has access to the core data supporting our conclusions. |
| 4. Discussion and interpretation  ϖ Recommendations:  - Deepen discussion of regional disparities and structural factors (access to care, infrastructure). - Incorporate consideration of the limitations of RDTs as a diagnostic tool (sensitivity, specificity). | While we agree that these points are crucial for a comprehensive study, we cannot introduce information because of the available data.  Having your Recommendations we added a section of Limitations and Future Directions to the discussion that acknowledges these limitations as areas for future research. |
| **5. Review Comments to the Author**  **Reviewer #2:** | |
| Reviewer #2: Additionally, the authors should correct various typographical and grammatical errors throughout the manuscript to improve the clarity and professionalism of the scientific writing. Special attention to:  1. Missing space after period at the end of a sentence, occurring in several sentences of the text. 2. "known as a vector of infection" could be simplified to "which acts as a vector". 3. "a bite from an infected insect". It is better as "a bite from an infected mosquito". 4. "This is compared to 593,000 cases in 2021" refers to deaths but uses "cases" erroneously - should be "deaths".  5. "children under five at particularly high risk" should be "children under five years old are at particularly high risk".  6. "[?, 12, 28]" The question mark indicates a missing or placeholder reference; it needs to be replaced with the proper citation number.  7. "Niger malaria indicator survey (NMIS)" - "Niger Malaria Indicator Survey (NMIS)"  (capitalize proper nouns).  8. "geographic coordinates (longitude and latitude)" - Standard order is "latitude and longitude." 9. "Enumeration Areas (EAs) or cluster level" - Should be "Enumeration Areas (EAs) or cluster level" or "clusters".  10. "Correct capitalization and style: "Stata v14".  11. Missing space after comma: "ICF International, the datasets".  12. "accessible web-site" — "website" is one word; hyphen unnecessary.  13. "write the studys title and signifi-cance" — Should be "study’s title and significance" with an apostrophe; hyphenation in "signifi-cance" should be corrected to "significance".  14. "Independent Variables The Geo-statistical model utilized individual and community-level variables 5.” – What is this 5?  15. Hyphenation & spacing: Several places require adding spaces after punctuation marks before continuing, e.g., after commas and periods.  16. Consider defining acronyms upon first usage if not done elsewhere, e.g., "RDT," "SaTScan," "ICC," "VA," "PCV," etc.  17. “but "47% (527)" says "female child yielded" where "child" should be plural "children". 18. "Out of the 4724 RDT tests conducted, 53% (594) were performed on male children, and 47% (527) of female child yielded positive results for malaria." Should be: "...and 47% (527) of  female children yielded positive.  19. "The tails bright red and blue colours indicate a higher level of significance (Fig 1 )." - Remove extra space before parentheses. "Globally Morans I=0.434025 (z-score=10.471517, p value 0.001)." Should be: "Global Moran's I = 0.434 (z-score = 10.47, p = 0.001)." Use apostrophe in "Moran's". | This level of comprehensive feedback is highly appreciated as it directly contributes to strengthening our work. We have carefully reviewed our writing and spaces after punctuations. All suggested changes, which we agree will significantly enhance the quality of our paper. Most of those mistakes happened because we are writing in LaTeX.   - We give definition for acronyms under the Acronym section.   VA is the variance of the initial model and VB is the variance of the model with additional terms. The description is given in methodology section. |
