# Supplementary material for: Exploring spatial variation and multilevel modeling of malaria prevalence among children aged 6-59 months based on RDT in Niger: Insights for public health decision-making
Source: PLoS One. 2025 Dec 1;20(12):e0336022. doi: 10.1371/journal.pone.0336022 (PMC12668621; doi:10.1371/journal.pone.0336022)
Supplement: Attachment — Submitted filename: Response_to_Reviewers_auresp_2.docx [file pone.0336022.s003.docx]

PONE-D-25-18518

**Exploring Spatial Variation and Multilevel Modeling of Malaria Prevalence among Children Aged 6-59 Months Based on RDT in Niger: Findings from the 2021 NMIS**

**PLOS ONE**

Manuscript Comment and Correction

**Response to Reviewer #1**

We sincerely thank the reviewer for the thoughtful and constructive feedback, as well as for recognizing the scientific contribution, methodological rigor, and overall quality of our manuscript. We appreciate the recommendation for *“acceptance after minor revision”* and are pleased to address each suggestion in detail below. All corresponding changes have been incorporated into the revised manuscript.

**Summary of Revisions Based on Reviewer Suggestions**

| **Reviewer Comment** | **Our Response** |
| --- | --- |
| **Title & Abstract – highlight practical relevance** | The title is modified and We added a sentence in the abstract underscoring the regulatory and public health significance of the findings. |
| **Introduction – add international regulatory context** | A brief overview of relevant global frameworks (WHO) has been included to broaden scope and applicability. |
| **Methodology – address bias and quality control standards** | We expanded the section on how the selection has done. |
| **Discussion – connect findings to pharmaceutical policy and normative frameworks** | Even if it is not enough, we tried to modify the discussion section. |
| **Conclusion – reinforce relevance to low-resource or African regulatory contexts** | The conclusion now includes a focused statement on the implications for regulatory systems in low- and middle-income settings, particularly within Africa. |

We appreciate the reviewer’s positive evaluation and constructive recommendations, which have significantly strengthened the clarity, regulatory relevance, and overall impact of the manuscript. We hope that the revisions satisfactorily address all concerns and respectfully submit the updated version for final consideration.
